# Right and left-sided colon cancers - specificity of molecular mechanisms in tumorigenesis and progression

**DOI:** 10.1186/s12885-020-06784-7

**Published:** 2020-04-15

**Authors:** Kavitha Mukund, Natalia Syulyukina, Sonia Ramamoorthy, Shankar Subramaniam

**Affiliations:** 1 Department of Bioengineering, University of California, San Diego, La Jolla, CA USA; 2 Division of Colon and Rectal Surgery, Moores Cancer Center, University of California San Diego Health System, La Jolla, CA USA; 3 Department of Cellular and Molecular Medicine, University of California, San Diego, La Jolla, CA USA; 4 Department of Computer Science and Engineering, University of California, San Diego, La Jolla, CA USA

**Keywords:** Left-sided colon cancer, Right-sided colon cancer, Biomarkers, Methylation, miRNA, RNA binding proteins, Xenobiotic metabolism, GPCR signaling, Non-synchronous tumors

## Abstract

**Background:**

Given the differences in embryonic origin, vascular and nervous supplies, microbiotic burden, and main physiological functions of left and right colons, tumor location is increasingly suggested to dictate tumor behavior affecting pathology, progression and prognosis. Right-sided colon cancers arise in the cecum, ascending colon, hepatic flexure and/or transverse colon, while left-sided colon cancers arise in the splenic flexure, descending, and/or sigmoid colon. In contrast to prior reports, we attempt to delineate programs of tumorigenesis independently for each side.

**Methods:**

Four hundred and eleven samples were extracted from The Cancer Genome Atlas-COAD cohort, based on a conservative sample inclusion criterion. Each side was independently analyzed with respect to their respective normal tissue, at the level of transcription, post-transcription, miRNA control and methylation in both a stage specific and stage-agnostic manner.

**Results:**

Our results indicate a suppression of enzymes involved in various stages of carcinogen breakdown including *CYP2C8*, *CYP4F12*, *GSTA1*, and *UGT1A* within right colon tumors. This implies its reduced capacity to detoxify carcinogens, contributing to a genotoxic tumor environment, and subsequently a more aggressive phenotype. Additionally, we highlight a crucial nexus between calcium homeostasis (sensing, mobilization and absorption) and immune/GPCR signaling within left-sided tumors, possibly contributing to its reduced proliferative and metastatic potential. Interestingly, two genes *SLC6A4* and *HOXB13* show opposing regulatory trends within right and left tumors. Post-transcriptional regulation mediated by both RNA-binding proteins (e.g. *NKRF* (in left) and *MSI2* (in right)) and miRNAs (e.g. miR-29a (in left); miR-155, miR181-d, miR-576 and miR23a (in right)) appear to exhibit side-specificity in control of their target transcripts and is pronounced in right colon tumors. Additionally, methylation results depict location-specific differences, with increased hypomethylation in open seas within left tumors, and increased hypermethylation of CpG islands within right tumors.

**Conclusions:**

Differences in molecular mechanisms captured here highlight distinctions in tumorigenesis and progression between left and right colon tumors, which will serve as the basis for future studies, influencing the efficacies of existing and future diagnostic, prognostic and therapeutic interventions.

## Background

Colorectal cancer (CRC) is a heterogeneous disease with distinct clinical, molecular, and pathophysiological characteristics. The Surveillance, Epidemiology and End Result (SEER) program of the National Cancer Institute, in 2018, identified CRC as the fourth most prevalent cancer, and the third leading cause of mortality due to cancers, with an average five-year survival of 64.5%. Traditionally, patient subgroups in CRC are associated with dysfunction of mismatch repair genes (microsatellite instable group, MSI); *KRAS*, *APC* and/or *BRAF* mutations (chromosomal instability group) and hypermethylation (CpG island methylation phenotype, CIMP) [1]. However, increasing evidence on the heterogeneity of these genetic and epigenetic changes, necessitated a model for identifying consensus molecular subtypes (established in 2015) [2]. It is now widely acknowledged that the heterogeneity extends beyond the above recognized molecular mechanisms. Location of tumor within the colon is gaining traction as crucial factor in determining disease progression, prognosis and management, and begs the question if colon and rectal cancers can be treated as being mechanistically similar [3]. To this extent we focus our attention on discerning the molecular mechanisms governing colon cancer, particularly tumors arising in the left and right colons.

Right-sided colon cancers (RSCC or proximal tumors) occur in the cecum, ascending colon, hepatic flexure and/or transverse colon, while left-sided colon cancers (LSCC or distal tumors) arise in the splenic flexure, descending colon, and/or sigmoid colon. This distinction has been observed at physiological, molecular and therapeutic levels for RSCC and LSCC [4]. For example, from the perspective of disease management, National Comprehensive Cancer Network (NCCN) Clinical Practice Guidelines in Oncology outline differences in therapeutic sensitivities of targeted drugs differ between sites in CRC for e.g. EGFR inhibitors, such as cetuximab, panitumumab, have been indicated to be less effective as first-line therapy for RSCC metastatic disease [5]. At a physiological level, RSCC patients are more likely to exhibit advanced tumor stage, increased tumor sizes, frequently poorly differentiated tumors, with increased lymphovascular invasion than LSCC patients [6]. Systematic reviews and meta-analyses have correlated RSCC with poor prognosis and overall survival [7]. At a molecular level, multiomics analyses have identified differences between RSCC and LSCC, including differences in miRNA control and transcriptional regulation and its immune landscape [8]. RSCC also tend to exhibit different mutational burdens and increased hypermethylation compared to LSCC [9].

To the best of our knowledge, recent studies focusing on molecular differences between RSCC and LSCC, have done so by directly comparing the two etiologies (e.g. [10]). However, tumor transforms the state of healthy tissue, and we would benefit by comparing tumor tissue with its normal first, and then extend the understanding to side specific differences in the etiology of colon cancer. The analyses outlined in this paper is grounded in this simple idea and focuses on mechanistic differences leading to the pathogenesis of these two cancer types. Towards this, we perform an integrative analysis using publicly available colon cancer (COAD) data (from non-synchronous tumor patients) in The Cancer Genome Atlas (TCGA) project. We first establish transcriptional regulation in both RSCC and LSCC, independently, compared to their respective normal tissue and identify common and distinct programs of regulation that exist between proximal and distal tumorigenesis. We next systematically identify and characterize roles for miRNAs and RNA binding proteins, in colon cancer, in a side specific manner. Differences in epigenetic (DNA methylation) profiles, and its impact on the observed gene regulation, within both of these colon cancer types are also comprehensively analyzed.

## Methods

### Sample inclusion criteria

TCGA level 3 COAD raw count data for 499 solid tumor and normal tissue was downloaded via Firebrowse in July 2018. Samples were classified into right/proximal and left/distal based on their site of extraction. Since TCGA clinical data was not specifically annotated for exact location along the transverse colon, all samples annotated “transverse colon” were excluded from analysis [11]. Samples with discrepancy in site of extraction, and with a history of “synchronous” colon tumors and/or adjuvant therapy were also excluded. 411 samples (170 LSCC and 17 Left normal and 203 RSCC and 21 Right normal) were subsequently utilized for RNAseq analysis. Baseline characteristics of the sample patient cohort is outlined in Supplementary Table S1.

### RNAseq analyses

Count matrix normalization and differential analysis was performed using the “DEseq2” package available through R/Bioconductor [12]. Expression of 17,597 genes was used as input after basic prefiltering. Differentially expressed genes (DEGs) for each side were identified at |log2| fold change (fc) > 1 and adj p-val < 0.05 (Benjamini-Hochberg). We reasoned that the thresholds chosen here were optimal to identify biologically relevant targets for the purposes of this analysis (Supplementary Table S2). Stage specific analysis (utilizing AJCC staging information extracted from the TCGA-COAD clinical metadata) was also likewise performed (Supplementary Tables S3 and S4). High confidence (> 0.9) protein-protein interactions (PPI) was downloaded from STRING database (v10.5). Side specific PPIs were extracted corresponding to DEGs on each side. Co-expression between genes was utilized as weights to cluster side-specific networks using MCL clustering. The mSVM-RFE method with 10 fold cross-validation [13] was utilized to identify DEGs, that improved classification efficiency of side-specific tumors from normal tissue (Supplementary Table S5).

### Cox regression analyses

Survival outcome modeled results with reference to patient overall survival (OS). Specifically, events were defined as death by any cause, and time was accurate to the day. *p*-values were obtained from univariate Cox proportional-hazards regression models for the entire list of differentially expressed genes on the right and left (using the R packages Regparallel and Survival). All Kaplan–Meier survival curves were constructed using survival and ggplots2 packages. Median expression was utilized to separate high from low expression group in left and right samples. A total of 76 and 42 gene markers were identified to be associated with overall survival in left and right respectively (logrank p-val < 0.01; Supplementary Table S6).

### miRNA analyses

miRNA expression data was downloaded using TCGAbiolinks [14], across 449 annotated samples (see Methods), of which a total of 370 patient samples met the inclusion criteria. Differentially expressed miRNAs (DEMs) for all pairwise comparisons were identified using DESeq2 (adj *p* < 0.05). *Manually* curated miRNA-target interactions from miRTarBase 7.0 [15] between DEGs and DEMs were extracted. Subsequently the left network contained 250 connected nodes, with 211 interactions (Supplementary Figure 1) and the right contained 319 connected nodes with 225 interactions (Supplementary Figure 2). Spearman rank correlation between miRNA and mRNA expression in tumor samples was added as edge weights. The networks were clustered on these weights (using MCL clustering) to ascertain clusters relevant to RSCC and LSCC.

### Quantification of differential alternative splicing

Percent splice-in (PSI) values were downloaded from TCGASpliceSeq (with PSI values in at least 75 samples) for our sample cohort, accounting for a total of 23,176 events. “psichomics” [16] available through Bioconductor/R was utilized to extract significantly differentially alternatively spliced (AS) events between tumor and normal samples for each side independently. Seven different event types were detected between normal and tumor samples for each side namely, Exon Skip (ES), Alternate Donor Sites (AD), Alternate Acceptor Sites (AAs), Retained Intron (RI), Alternate Promoter (AP), Alternate Terminator (AT) and Mutually Exclusive Exons (ME) [17]. Significantly differentially AS events were defined as events with a |Δ median PSI| ≥ 0.1 and FDR ≤ 0.01 (Supplementary table S10) [16]. Of all the events detected, only events occurring in DEGs were considered significant for our current analysis and chosen for further study (henceforth referred to as “sigAS” events).

### Identifying key RNA-binding proteins

A combined list of 1608 RNA-binding proteins (RBPs) were extracted from supplementary tables available in two prior publications [18, 19], consolidating known RBPs. We referenced this RBP list against the list of DEGs identified in LSCC and RSCC to identify differentially regulated RBPs (log2 fc > 1, adj *p* < 0.05) for each side independently. We utilized the entire list of DEGs on each side to identify enrichment of RBP binding using AURA2 [20]. Since, we were particularly interested in RBPs that contribute to sigAS, we extracted 295 splicing associated RBPs (with GO RNA related category description containing keyword “splicing” from [19]). 4 and 5 RBPs were identified to be differentially regulated in RSCC and LSCC, respectively, and were considered for further analysis. Spearman correlation between expression (transcripts per million, downloaded from Firebrowse as before) of the differentially expressed splicing associated RBPs against PSI values for sigAS events was performed within tumor samples, on left and right. Clustering was performed using MCL clustering.

### Methylation analyses

Illumina HumanMethylation450 beadchip data for TCGA-COAD, probing nearly 480 k CpG sites, was downloaded using TCGAbiolinks. Nearly 370 k probes were retained for each side, after eliminating probes on sex and “NA” chromosomes. Mean methylation and differentially methylated probes (DMPs) were both determined using the TCGAbiolinks package, independently for each side. DMPs were identified using *β (average promoter methylation value %)* that were at least 25% differential (|Δ*β*| > 0.25) between tumor and adjacent normal tissues with adj *p* ≤ 0.001, for each side. Correlated and anti-correlated pairs of DEGs with DMPs (Hypo and Hyper) and transcription factor (TF) enrichment were identified using ELMER [21].

### Enrichment and visualization

All functional enrichment analyses were performed using mSigDB’s hallmark data sets and GO ontologies. All networks were constructed, clustered, annotated and analyzed in Cytoscape [22]; Visualization was performed using clusterProfiler [23]. Transcription factor enrichment of targets was performed using ARCH^4^ database [24] or ELMER where applicable.

## Results

### Establishing transcriptional regulation in RSCC and LSCC

**Fig. 1:**
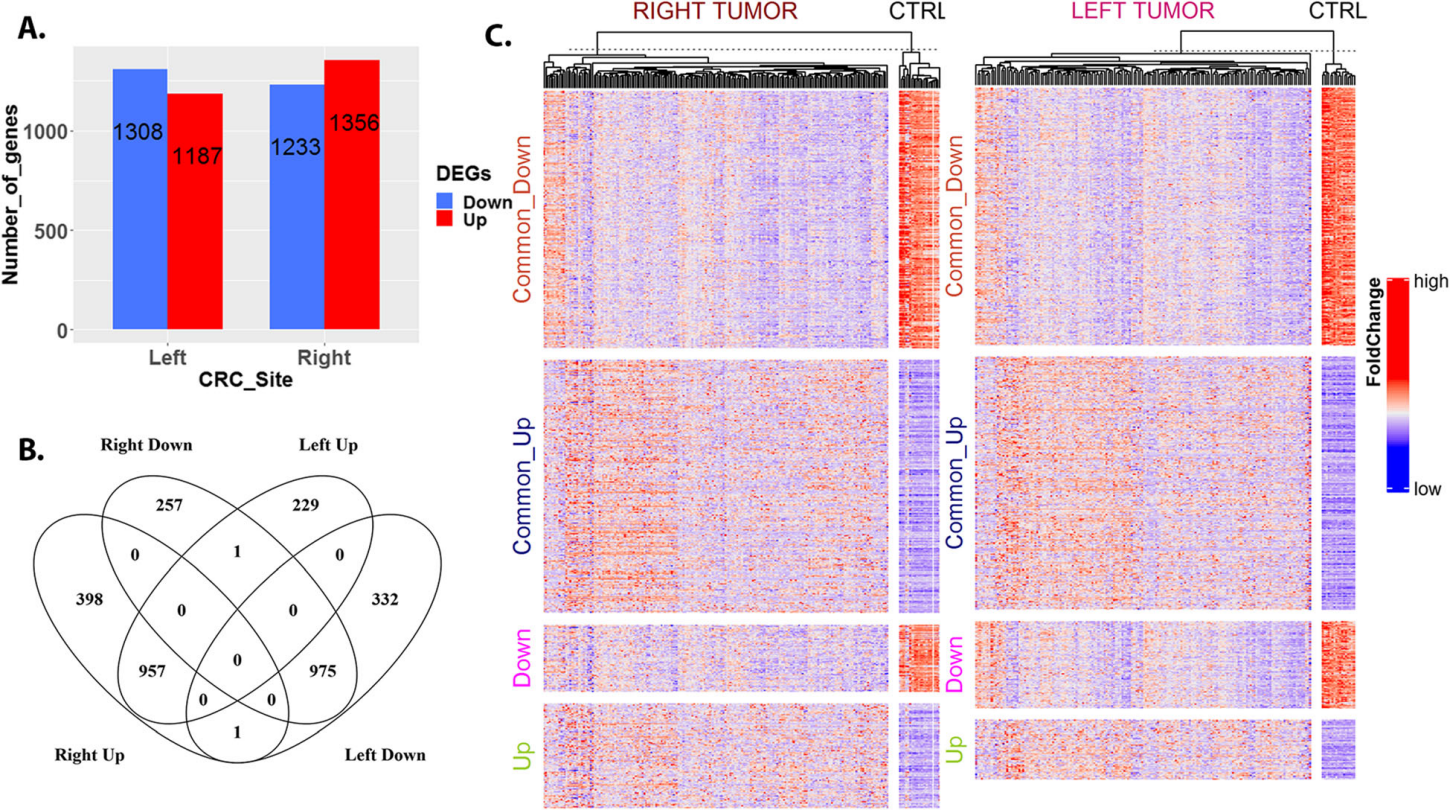
Transcriptional signatures of right and left-sided colon cancers. **a** The distribution of differentially regulated genes (DEGs, up and down) identified within LSCC and RSCC, compared to their respective normal. **b**. The overlap between RSCC and LSCC DEGs identifying commonly and uniquely regulated genes **c**. A heatmap of the gene expression for both LSCC and RSCC samples, highlighting expression features of commonly dysregulated (Common_Up and Common_Down) and uniquely dysregulated (Up and Down) genes sets within the left or right colon, compared to their respective normal tissue

Differential analysis identified 2495 DEGs in LSCC, with respect to left normal colon tissue, and 2589 DEGs in RSCC, with respect to right normal colon tissue (Fig. 1a). 957 (up) and 975 (down) DEGs are identified to be commonly regulated in both RSCC and LSCC while, 655 and 561 DEGs are “uniquely” regulated in RSCC and LSCC, respectively (Fig. 1b and c).

### A common program of tumorigenesis exists between right and left sided colon tumors

**Fig. 2:**
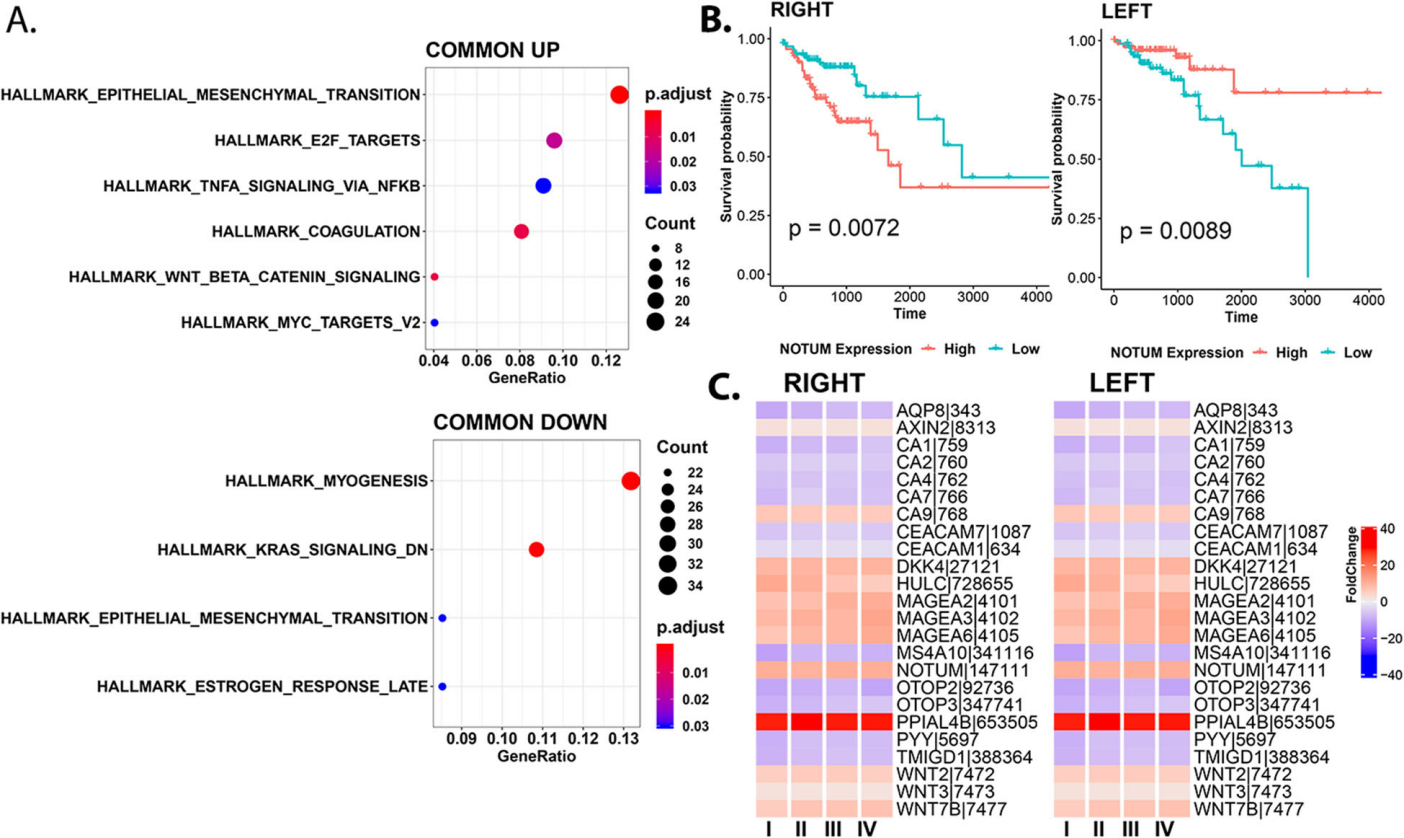
Transcriptional landscape of commonly regulated genes. **a**. Enrichment of Hallmark gene sets from mSigDB, for genes commonly regulated in both LSCC and RSCC. **b**. Overall survival associated with *NOTUM* expression- Kaplan-Meir curves capture the association between NOTUM expression and overall survival of patients with RSCC and LSCC. **c**. Expression fold changes captured for commonly regulated genes in both LSCC and RSCC, across all four stages 1–4 indicated as T1-T4

Malignant tumor cells are highly plastic and are characterized by alterations in metabolism, adhesion, proliferation and migration, requiring coordinated activity of several signaling pathways and mechanisms. Our results indicate that the large overlap of “commonly” regulated DEGs that exist between RSCC and LSCC fit broadly into these categories and are frequently seen dysregulated in colon cancers (Fig. 2a). For instance, dysregulation of WNT/β-catenin pathway genes affecting proliferative potential of CRC is evident in both, with the upregulation of several WNT pathway genes including *AXIN2, WNT2, WNT3, WNT7B, DKK1/4, NKD1/4, TCF7, MYC* and *NOTUM*. *NOTUM*, a glypican-dependent WNT inhibitor serves as a negative feedback regulator for WNT activation [25], and is associated with the progression of CRC [26]. We identified *NOTUM* to be significantly associated with OS in patients with both RSCC (Hazard Ratio 95% CI - 0.44 (0.24–0.82), logrank *p* < 0.01) and LSCC (HR 95% CI – 3.23 (1.27–8.2), p < 0.01) indicating that a higher expression favors LSCC while lower expression favors RSCC (Fig. 2b). Other frequently dysregulated genes including *APC*, *GSK3B* were identified as commonly dysregulated (albeit below our fc threshold).

Cellular metabolism is tightly linked with cellular growth and proliferation of tumors. Loss of AMP-activated protein kinase (*AMPK1*) activity can drive reprogramming of cellular metabolism and is at the center of the network regulating cell growth and proliferation (via *TP53*). *AMPK1* driven-metabolic dis-homeostasis becomes evident by the observed dysregulation of melanoma antigens (*MAGEA2/A3/A6*) [27], within both these tumor types. The acidic and hypoxic tumor microenvironment also influences survival and proliferative potential. Carbonic anhydrases (CA), metalloenzymes which catalyze reversible hydration of CO_2_, have been identified as crucial mediators of tumor pH [28]. Notably, several cytosolic CAs (such as *CA1/2/4*) and water channels (*AQP8*) are suppressed in both, alluding to the reduced availability of the universal buffer HCO_3_^−^. It has been previously postulated that extracellular CAs such as *CA9* (upregulated in both tumor types), act to raise the extracellular pH favoring tumor cell growth, proliferation, and survival [29]. Several other markers including FDA approved CRC biomarkers such as CA125 (*MUC16*) and CEA (carcino-embyonic antigen, *CEACAM1/7*) are upregulated in both RSCC and LSCC.

Interestingly, several genes whose precise molecular interactions are yet to be completely understood, are among the most highly expressed genes in both proximal and distal tumors including *OTOP2* (controlled by wild-type *TP53*) [30], *OTOP3, PYY* and *PPIAL4*; and many with limited supporting evidence, might serve as interesting candidates for future research in colon cancers (Supplementary Table S7). Additionally, several genes discussed herein are regulated across all stages further highlighting their role in the evolution and maintenance of tumors over time, in a side-independent manner (Fig. 2c).

### Right-sided colon tumors exhibit altered lipid, bile and xenobiotic metabolism

**Fig. 3:**
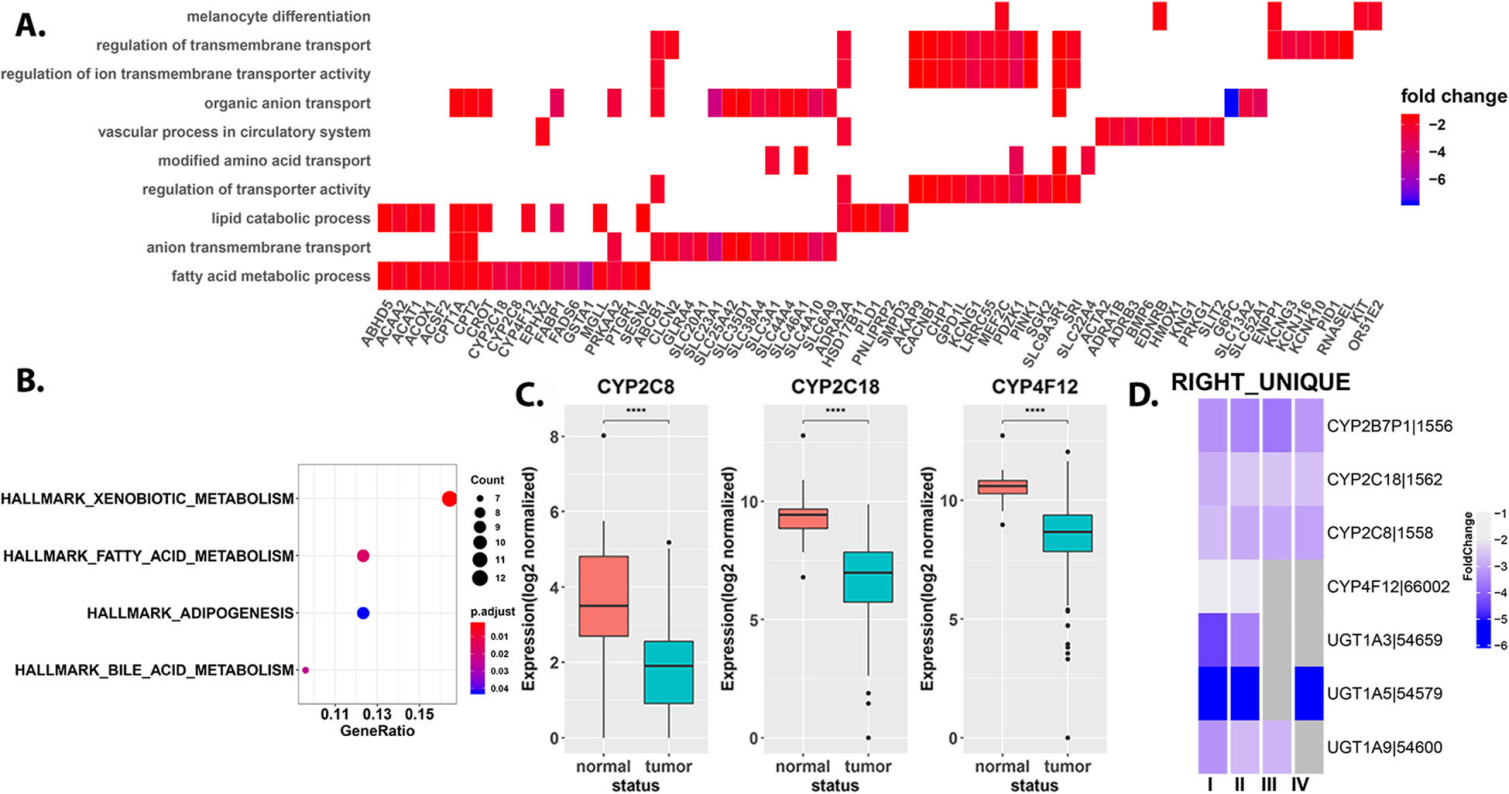
Right-colon specific transcriptomic (dys) regulation. **a**. Enrichment of hallmark gene sets within genes uniquely downregulated within RSCC identifies a marked suppression of genes particularly associated with metabolism (bile, xenobiotic and fatty acid). **b**. Box and whisker plots of gene expression counts (stage agnostic) for 3 genes (CYP2C8/18 and CYP4F12) suppressed in right tumors compared to their respective normal tissue. **c**. Stage specific expression (fold changes) for select genes discussed in main text associated with xenobiotic metabolism, captured across stages (T1-T4) in RSCC. Grey indicates non-significant expression (see Methods)

Liver is largely considered the major organ for biotransformation (chemical detoxification and metabolism). However, there is increasing acknowledgement of extra-hepatic biotransformation (especially in the gastrointestinal (GI) tract) and its association with GI carcinogenesis. Several families of enzymes are associated with various stages of breakdown of carcinogens within the human body including cytochrome P450 (CYP), glutathione S-transferase (*GSTA1*), and UDP-glucuronosyltransferase (UGT) superfamily [31]. Notably, we identify these gene families to be enriched among gene-sets suppressed in RSCC (Fig. 3a). Particularly, we identify a suppression of enzymes from CYP2C and 4F families (*CYP2C8, CYP2C18* and *CYP4F12*, Fig. 3b). These results are interesting in light of a recent study [32], which identified contrasting results with an upregulation of CYP2C family of enzymes in animal models of CRC. The CYP2C pathway enzymes convert arachidonic acid (AA) into active epoxyeicosatrienoic acids (EETs), while CYP4F family convert AA to hydroxyl EETs, both compounds suggested to promote carcinogenesis in certain contexts. UGT proteins catalyze the glucuronidation reaction, allowing for the utilization and/or detoxification of necessary chemicals [33]. We identify suppression of several UGT1A isoforms in tumor compared to normal, particularly the extra-hepatic isoforms *UGT1A10/A7 and A8*.

Proximal tumors predominantly exhibit dysregulation of UGT1A hepatic isoforms *UGT1A3* and *UGT1A9*. Several solute carrier transporters particularly associated with drug (*SLC25A42*, *SLC44A4*, *SLC46A1*) and ascorbic acid transport (*SLC23A1/A3*) are also suppressed in RSCC. Stage specific analysis revealed a unique dysregulation of members from the CYP2C and UGT1A family within proximal tumors, specifically at early stages (T1-T2) (Fig. 3c).

Cancer cells preferentially use aerobic glycolysis to metabolize glucose, over mitochondrial oxidative phosphorylation (OXPHOS) characterized by increased glycolysis and lactate production. Our results suggest a more pronounced shift in metabolism in proximal tumors over distal tumors. The selective upregulation of *SLC2A1* (GLUT1), a pivotal rate-limiting element in the transport and uptake of glucose combined with the unique downregulation of several mitochondrial metabolic markers involved in fatty acid degradation and oxidative phosphorylation including *G6PC, FABP1, CPT1A, CPT2, ACAT1, ACAA2, ACOX1, EPHX2* and *EHHADH* further support the more pronounced shift in metabolism away from OXPHOS, within primary proximal tumors, asserting its more aggressive state [34].

### HOXB13 and SLC6A4 show opposing regulation trends in RSCC and LSCC

**Fig. 4:**
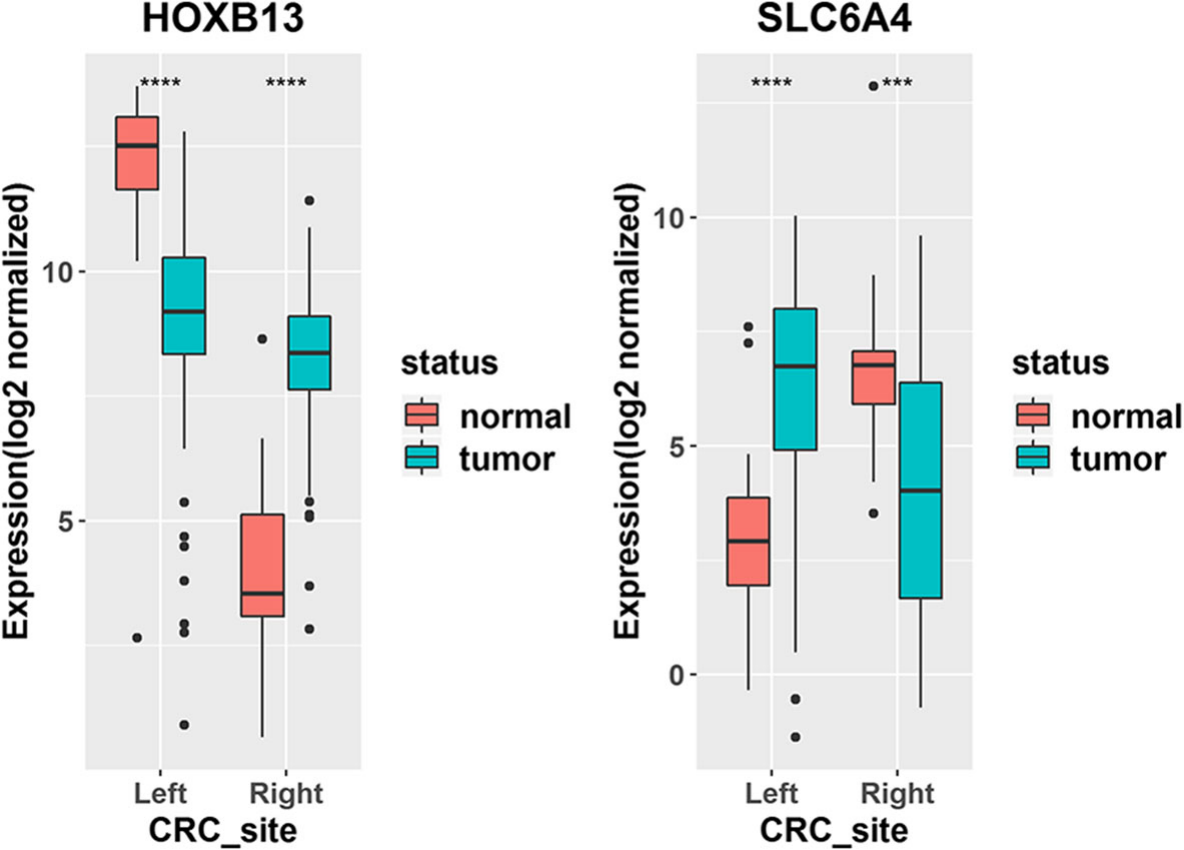
Expression counts of HOXB13 and SLC6A4, within proximal (right) and distal (left) tumors and their corresponding normal tissue

Two genes appear to exhibit opposing regulation trends within RSCC and LSCC – *HOXB13* and *SLC6A4* (Fig. 4). Nearly 95% of the body’s neurotransmitter-serotonin (5-hydroxytryptamine; 5-HT) is generated by the enterochromaffin cells, catalyzed by tryptophan hydroxylase (*TPH1/2*) within the intestine. Global loss-of-function studies for *TPH1* have indicated an almost complete loss of intestinal 5-HT synthesis, implying that the observed suppression of *TPH1* in both RSCC and LSCC indicates a curbed extracellular production of 5-HT [35]. Similarly, suppression of 5-HT receptors (e.g. *HTR3E, HTR4*) and intracellular enzymes required for breakdown of 5-HT (e.g. *MAOA, MAOB*) indicates a decreased bioavailability of 5-HT in both LSCC and RSCC. In light of this, it is reasonable to observe a suppression of *SLC6A4* (a ligand gated serotonin-selective reuptake transporter (SERT)), required for transport of 5-HT, such as in the case of RSCC. Though no evidence in literature exists for the differential role of *SLC6A4* in proximal or distal tumors within humans, we speculate that the observed upregulation of SERT expression within distal tumors (indicative of its increased activity), suggests alternate roles for *SLC6A4* and/or mechanisms controlling its expression within LSCC.

On the other hand, *HOXB13* is an acknowledged oncogene. Studies geared specifically towards specific tumor location, have identified suppression of HOXB13 within distal tumors [36] and upregulation within proximal tumors [37], consistent with our current analysis. Interestingly however, we also observe an upregulation of *PRAC1* and *PRAC2* (C17orf93), two genes genomically adjacent to *HOXB13*, within proximal tumors.

### Suppressed immune signaling predominates left-sided colon tumorigenesis

**Fig. 5:**
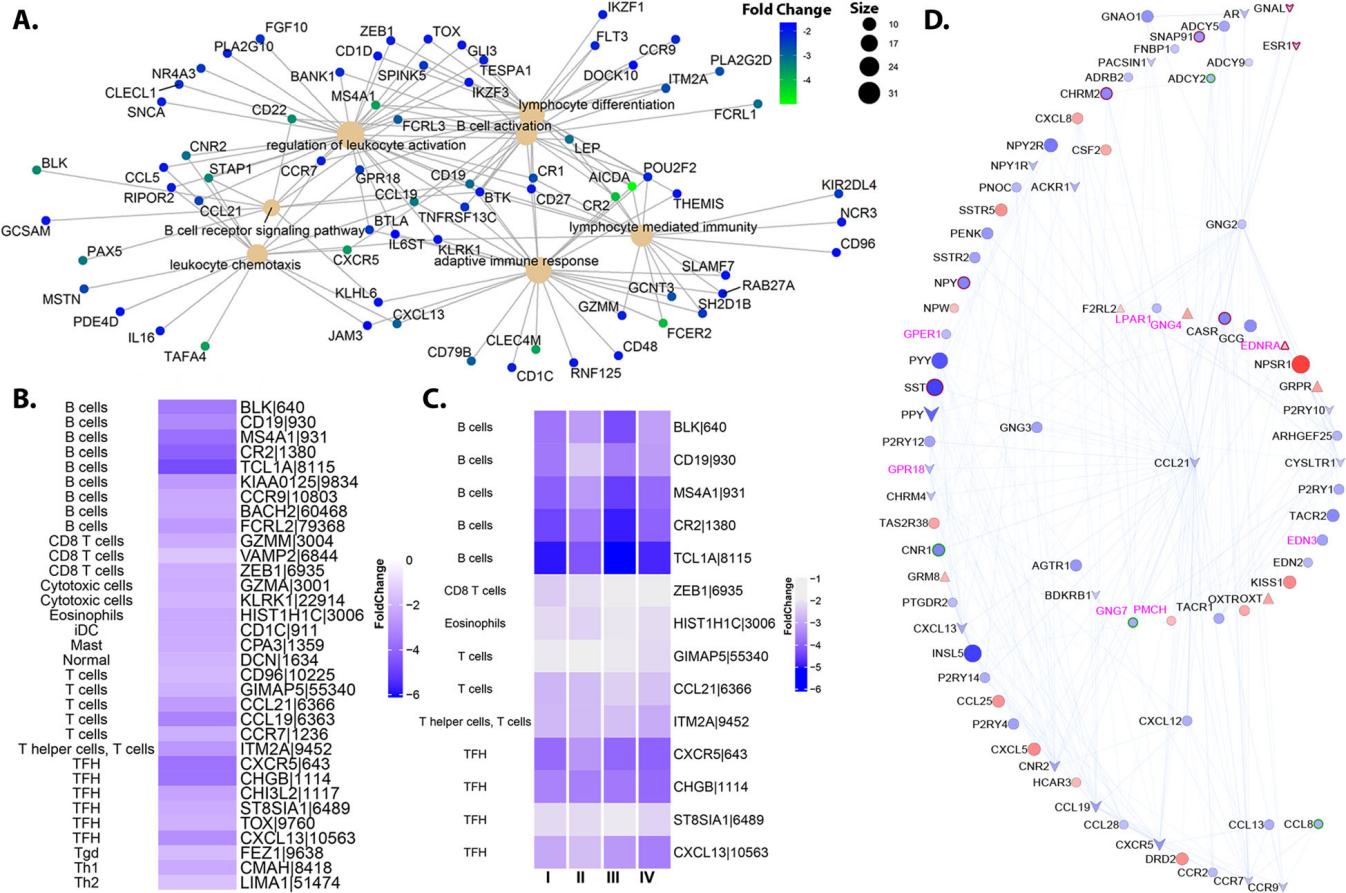
Left-colon specific transcriptomic (dys)regulation. **a**. A graphical representation of suppressed gene functions (based on Gene Ontology) and its associated genes. Color on the gene nodes indicate their fold changes (compared to Left normal tissue) and size of functional nodes indicate the number of genes associated with that function. **b**. Fold changes of immune markers that are dysregulated within LSCC (stage agnostic) **c**. Stage specific capture of fold changes for immune markers that were suppressed across all stages of LSCC. **d**. Protein interaction cluster (see Methods) which highlights the correlation patterns among GPCR and chemokines within LSCC. The color intensity is indicative of the strength of fold change going from light to dark (low to high fc) in either direction. Blue- downregulated, red- upregulated, triangle- uniquely upregulated, down arrow- uniquely downregulated, green border- hypomethylated, red border-hypermethylated

Chemokines are expressed by various cell types, constitutively or under inflammatory conditions. Remarkably, distal tumors exhibit an enrichment of suppressed chemokine signaling, particularly B-cell and TFH markers, important immune infiltrates in colon cancer (Fig. 5). The role of B-cells, and its supporting cell types in immunosurveillance is complex and dichotomous. On one hand, animal models studies suggest participation in proliferation and metastasis by promoting chronic inflammation, and suppressing antitumor responses [38], while on the other hand, promote long term survival leading to increased intratumor densities of tumor infiltrating immune cells suppressing tumorigenesis [39]. *MS4A1* (CD20, tumor infiltrating B-cell marker), and *BACH2* (a well-known transcriptional regulator of B and TFH cells), two genes previously implicated in contributing to immune landscape differences between RSCC and LSCC [39], are more predominantly suppressed within distal tumors. Particularly interesting is the predominant downregulation of two chemokine signaling axes, within distal tumors (compared to normal) including the homeostatic chemokines *CXCL13/CXCR5* (TFH cell markers) and *CCL19/CCL21- CCR7* (migration and activation of immune cell types). Several of these markers including *MS4A1, CXCR5,* and *CXCL13* are also suppressed across all stages (Stages 1–4) with respect to normal, further emphasizing their role in sustaining tumor behavior within LSCC (Fig. 5b and c).

In order to better understand the observed suppression of chemokine markers in a larger framework of regulation within distal tumors, we extracted clusters from a distal-specific protein interaction network (see Methods, Fig. 5d). We detected a large cluster of chemokines co-expressed with several G-protein couple receptor signaling and cAMP signaling proteins, including *GRM8* (a cell surface marker in CRC), *GNG2/4/7*, *EDN2/3* and *ADCY2/5/9* (adenylate cyclase). Two downregulated receptors *LPAR1* (Lysophoatidic acid receptor), and *CASR* (Ca^2+^ sensing receptor) involved in Ca^2+^ homeostasis, were also detected within this cluster [40, 41]. Taken together, these results lead us to speculate on a nexus between altered Ca^2+^ signaling mediated by GPCRs, specifically chemokines, and their subsequent impact on the inflammatory signatures within distal tumors (LSCC). Notably, 8/78 genes within this cluster including *LPAR1, GNG4, GNG7, PMCH, GPR18, EDNRA, GPER1* and *EDN3*, (hypergeometric *p* < 0.07), are sufficient to distinguish distal tumors, from normal distal colon as identified via recursive SVM classifier (see Methods).

### RSCC exhibits pronounced post-transcriptional regulation

Small non-translatable RNAs called miRNAs and several other RNA-binding proteins (RBPs), form an important class of molecules involved in post-transcriptional regulation (PTR). We focused on utilizing two levels of -omics data for analyzing differences in PTR within LSCC and RSCC.

#### Side-specific control of tumorigenesis by miRNAs

Side-specific differential analysis of 1046 micro-RNAs (miRs) identified 325 differentially regulated miRs in RSCC and 200 miRs in LSCC, compared to their respective normal tissue (see Methods, Supplementary table S8). A large majority of dysregulated miRs (198) are changing in both RSCC and LSCC. Several of the top commonly upregulated miRs are oncogenic-miRs including miR-135b, miR-577, miR-19a, miR-592 with roles in tumor initiation, proliferation/ progression and migration [42, 43]. Likewise, several miRs suppressed within both tumor types, including miR-328 [44], miR-486 [45], have been previously indicated in inhibition of tumor progression in CRC.

**Fig. 6:**
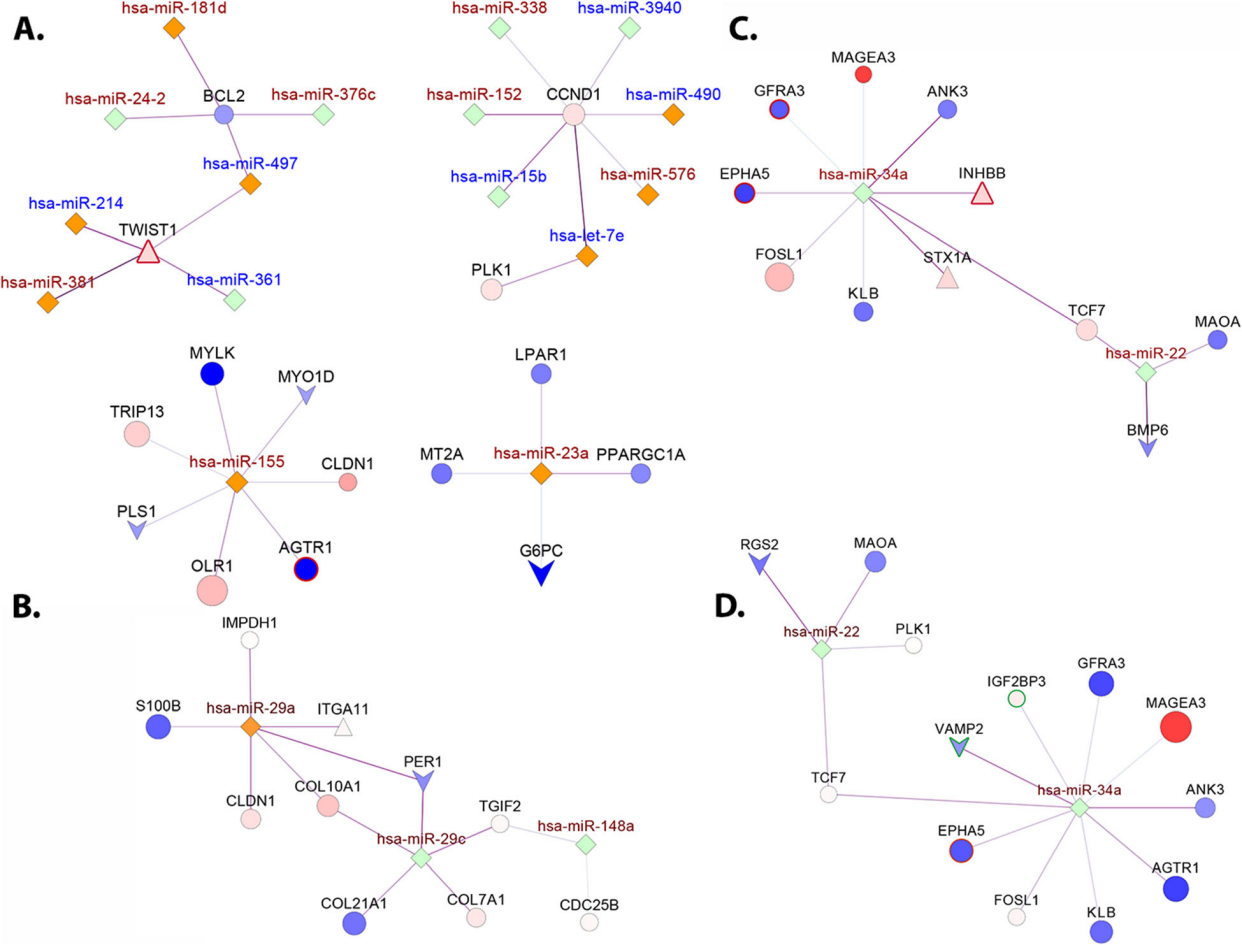
miRNA-mRNA interaction networks for right and left-sided colon cancers. **a**. Select clusters extracted from RSCC interaction network, with several uniquely regulated miRs (orange) are involved in regulating genes associated with cell cycle control. **b**. A cluster extracted from LSCC, containing the miR-29 (miR-29a and miR-29a) indicates a possible role for these miRNAs in regulating the interaction between period genes and ECM markers. **c** and **d** indicate the cluster containing miR-34a and miR-22 extracted for RSCC and LSCC respectively. Node color: Blue- downregulated, red- upregulated, green- commonly regulated miR and orange- uniquely regulated miR. miR label color: red- upregulated, blue- downregulated

Increasing evidence however suggests malleable roles for miRs, with multiple targets, amplifying their inhibitory or stimulatory effects on gene regulation through positive or negative feedback loops in conjunction with other miRs. We established functionally relevant, side-specific miRNA-mRNA clusters (see Methods) in an effort to identify the influence of the differentially regulated miRs on gene expression. Analysis of clusters within RSCC revealed miRs regulating genes in interconnected pathways of cellular metabolism, cell growth and proliferation (Fig. 6a). For instance, uniquely up-regulated miR-23a correlates with several mitochondrial proteins including *G6PC* and *PPARGC1*. Several miRs, particularly, miR-181d and miR-576, correlate with cell cycle genes including *BCL2* and *CCND1*. *BCL2*, a major regulator of mitochondrial apoptosis, has been consistently shown to be down regulated in colon (and cancer) [46]. Control of *BCL2* expression via miR-24-2 (strongly upregulated in both proximal and distal tumors), has been previously reported in human embryonic kidney and breast cancer cell lines [47]. Interestingly, several uniquely regulated miRs correlate significantly with (hypermethylated) *TWIST1*, a primal transcription factor uniquely upregulated within proximal tumors [48], whose activation has been implicated in reverting cells to a non-lineage specific proliferative state.

Only two miRs however, are uniquely regulated within distal tumors– miR-3607 and miR-29a (Fig. 6b). Interestingly, members of miR-29 family of oncomiRs (miR-29c and miR-29a) appeared to correlate with ECM and clock genes within distal tumors including downregulated *PER1* (negative regulator of circadian rhythm) [49].

Particularly interesting are clusters conserved within both RSCC and LSCC (Fig. 6c and d). For example, miR-22 and miR-34a, two commonly regulated miRs in CRC, appear to cluster together. These miRs are known to impinge on processes of metabolism, angiogenesis, proliferation, migration, invasion, apoptosis and epithelial-to-mesenchymal transition (EMT) (a primary transformation for metastatic and invasive tumor cells. miR-34a (a tumor suppressor induced by p53 involved in EMT in CRC) [50, 51] correlates with several commonly regulated genes involved in signal transduction and EMT via the WNT and AKT signaling pathways including *MAGEA3, GFRA3, EPHA5, ANK3* and *TCF7*. The uniquely upregulated *INHBB*, which correlates with miR-34a expression in proximal tumors (Fig. 6c), was also identified to be significantly associated with OS in RSCC (HR 95% CI - 0.34 (0.18–0.65), logrank *p* < 0.001).

#### Differences in alternative splicing events mediated by RNA-binding proteins in LSCC and RSCC

**Fig. 7:**
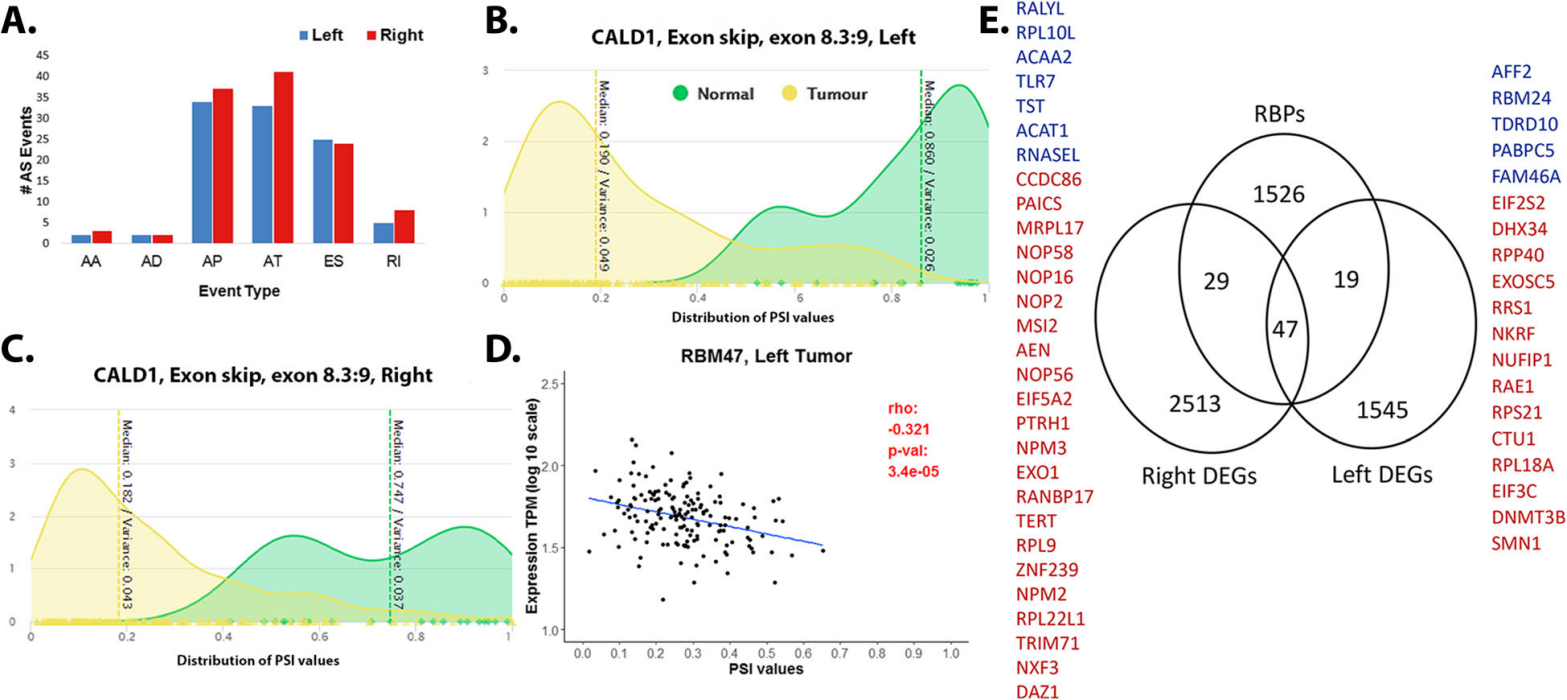
Summary of post-transcriptional regulation. **a**. Number of AS events identified in left and right among DEGs (sigAS) events belonging each of the six event types (AA- Alternate acceptor, AD- alternate donor, AP- Alternate promoter, AT- Alternate terminator, ES- Exon skip, RI- Retained Intron). **b** and **c**. Density plots for PSI value distributions of CALD1 gene in tumor and normal tissues for left and right respectively. D. Significant anti-correlation observed between expression of RBM47 (an RBP known to be suppressed in CRC) and the corresponding percent spliced-in (PSI) values within distal tumors. E. Venn highlighting RBPs identified among DEGs in LSCC and RSCC. RBPs identified as uniquely regulated on right (29 RBPs) and left (19 RBPs) are identified in color (blue-downregulated, red- upregulated)

Alternative splicing (AS) is an active PTR mechanism during which mRNA is actively rearranged accounting for the observed protein repertoire of complex organisms [17]. Utilizing Percent Splice-In (PSI) values from TCGASpliceSeq (see Methods), we identify 115 sigAS events among DEGs in RSCC and 101 sigAS events among DEGs in LSCC (see Methods). Exon skipping (ES), usage of alternate promoters (AP) and terminators (AT) were detected to be predominant and potent mechanisms for AS contributing to the etiology of colon cancers (Fig. 7a, Supplementary Table S10). Notably, our results indicate that a large proportion of the sigAS events (*n* = 64) occur in genes commonly dysregulated in both LSCC and RSCC, making alternative splicing a major PTR regulatory mechanism within colon cancers, including genes such as *AXIN2* (ES, exon 7), and *MXI1* (AP, exon 3) associated with the WNT pathway, and others such as *IGF2* (AP, exon5), *CXCL12* (AT, exon 5.2), *CCL24* (AP, exon 1), and *S100A2* (AP exon 3). *SULT1A2* (RI, 1.2:1.3) and *CALD1* (ES, 8.3:9) exhibit the highest Δmedian PSI values between healthy and tumor tissues, in both left and right. *SULT1A2* (suppressed ~ 3 log2 fc in both right and left tumors) is a sulfotransferase liver enzyme involved in detoxification of a variety of endogenous and xenobiotic compounds [52], while *CALD1* is a novel target of TEA domain family member 4 involved in cell proliferation and migration (Fig. 7b and c). Missplicing of both these transcripts have been previously detected as events correlated with the etiology of disease [53, 54]. Particularly interesting are sigAS events that occur in a side specific manner, within genes uniquely regulated in either RSCC or LSCC, for instance, *CYP4F12* (Δmedian PSI = − 0.19), *UGT1A1* (Δmedian PSI = 0.12) *SRI* (Δmedian PSI = − 0.64) all exhibit significant AS within right tumors.

We identify a total of 76 and 66 RBPs to be differentially regulated in RSCC and LSCC, respectively. A large proportion (47 RBPs) are commonly regulated in both RSCC and LSCC with several enriched for binding among DEGs (adj *p* < 0.05, see Methods), including RBPs previously discussed in the context of CRC such *MSI2, MEX3A, IGF2BP1/3, ELVAL4* [55, 56], and cancers in general, such as *RBM47*, *DKC1, CELF4, ELAVL3* (Fig. 7e, Supplementary Table S11). Downregulation of *RBM47* is involved in increased cell migration and invasion, and is indicated to promote EMT and metastasis within CRCs [57]. Notably, *RBM47* is also significantly differentially spliced within both distal and proximal tumors, compared to normal tissues (AP, exon 2). However, a significant anti-correlation between its expression and PSI values is observed only within distal tumors (Fig. 7d), implying a possibility of feedback mechanisms controlling *RBM47* within distal tumors.

Additionally, we identify significant correlation between the expression of *CELF4, RBM20, NOVA1* and *PPARGC1A* splicing associated RBPs and sigAS events in both proximal and distal tumors (see Methods). *AFF2* is however uniquely associated within distal tumors. The resulting correlation network indicated that greater than 50% of sigAS events (66/101-left and 58/115) are correlated with these specific RBPs (adj *p* < 0.05, Supplementary Figure 3), highlighting a possibly crucial role for them in the observed (many-to-one) regulation of transcripts within colon cancer.

### Differences in marker methylation and its association with gene expression, in RSCC and LSCC

**Fig. 8:**
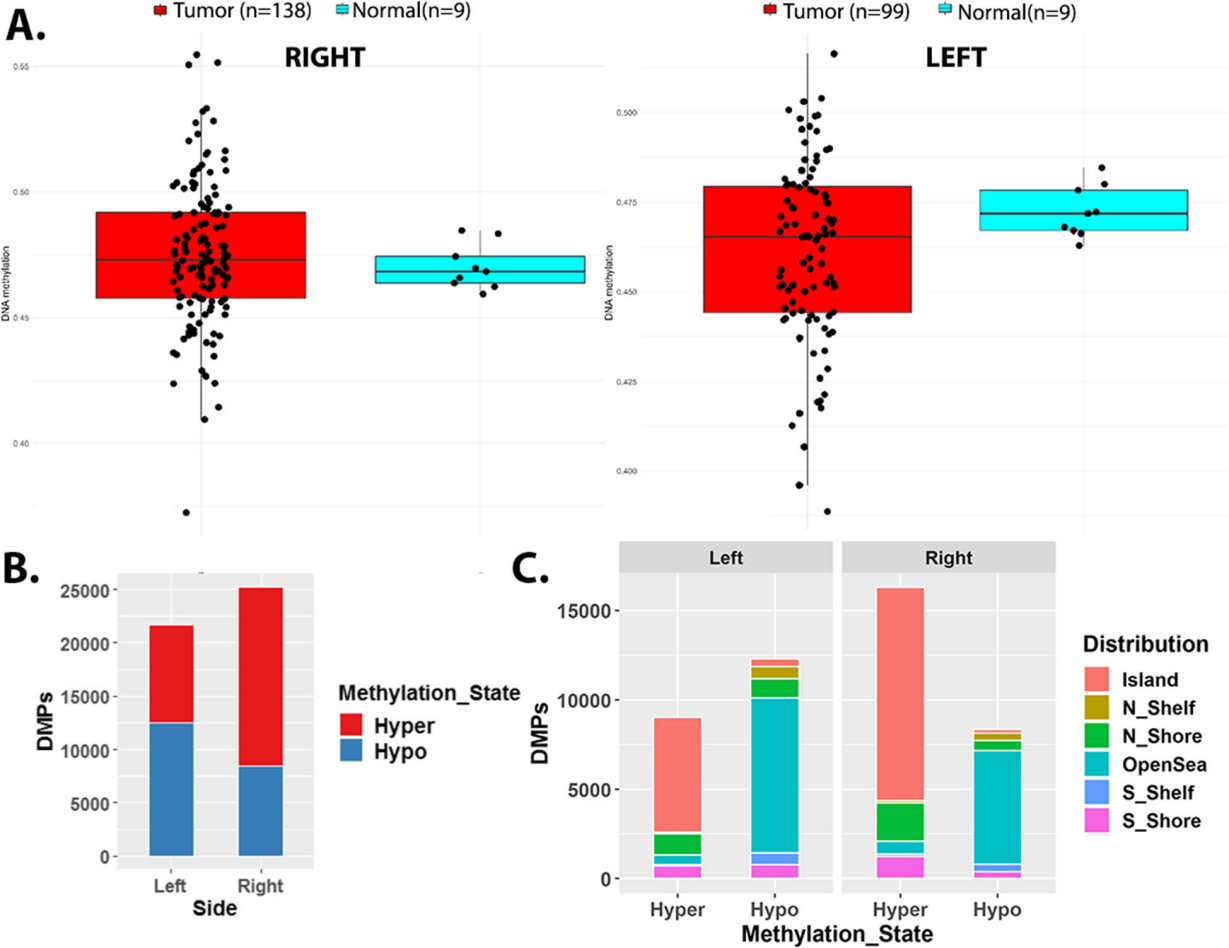
Right and left-sided colon cancers exhibit differential methylation patterns. **a**. Mean methylation (y-axis) of tumor and normal samples from the right colon and left colon respectively. **b**. Counts of differentially (hypo and hyper) methylated probes (DMPs) identified in left /LSCC and right colon/RSCC. **c**. Genomic distribution of the DMPs identified in both LSCC and RSCC, islands represent the annotated CpG islands, shores are 2Kb upstream/downstream from the end of islands. Shelves are 2Kb upstream/downstream of the farthest upstream/downstream limits of the shores with the remaining genomic regions making up the open seas

Development and progression of colorectal cancer is understood to undergo several genetic and epigenetic changes. Changes in the DNA methylation is one of major epigenetic mechanisms controlling CRC [9]. Differential methylation analysis identified a larger proportion of hypermethylated CpG sites (DMPs) in proximal/RSCC samples; while distal/LSCC exhibited a larger proportion of hypomethylated sites, compared to their controls respectively (see Methods, Fig. 8a). It is interesting to observe the genomic distribution indicated highest number of hypermethylated DMPs within the CpG Islands, while hypomethylation occurs in Open Seas (Fig. 8b). Previously published methylation markers including *SEPT9, VIM, GATA4, INA, MAL, WNT (WNT2/2B/3/6/5A/7A, APC2)* and *CNRIP1* are hypermethylated in both RSCC and LSCC [58, 59], further establishing them as side-agnostic methylation markers of CRC.

We were additionally interested in identifying impact of differential methylation on DEGs and to this extent, extracted significant probe-gene pairs (both anti-correlated and correlated) from both RSCC and LSCC. We find that 33% of the downregulated genes are significantly anti-correlated with at least one hypermethylated probe and 27% with hypomethylated probes within distal tumors. On the other hand, we found a higher fraction of genes being controlled by differential methylation in proximal tumors (~ 40% of the downregulated/hypermethylated genes, and ~ 20% upregulated/hypomethylated genes) indicative of a role for increased hypermethylation in suppressing expression with RSCC, consistent with prior research. Interestingly however, the hypermethylation and expression states of several commonly regulated DEGs (such as *OTOP2/3, CA1/2/4, NOTUM*) is more obvious in LSCC than RSCC (Supplementary Table S9). Notably, we identified a significant enrichment of gene-probe pairs that exhibited positive correlation (overexpression and hypermethylation in tumors). For instance, *WNT5A/2/3/7B* all exhibit significant correlation between expression and methylated DMPs in LSCC.

Changes in methylation state of region can be due to gain/loss of site-specific transcription factors [60]. We employed ELMER, to obtain insight into motifs and TFs which may be involved in setting tumor specific DNA methylation patterns within LSCC and RSCC (see Methods). In both these tumor types, we identify the *FOSL1* binding motif to be highly ranked for hypomethylated/upregulated loci, indicating a possible gain of *FOSL1* (significantly up ~ 3 fc, in both), in a side independent manner. Likewise, downregulated/hypermethylated loci are enriched for the *SP1/2/3* binding motifs. TF factors including *ISX* (suppressed ~ 2.3 fc in both), contain these binding motifs, suggesting an observed loss of these site-specific TFs might dictate de-novo hypermethylation and suppression of its downstream targets, in a side independent manner within colon cancers.

## Discussion

Proximal and distal colon tumors are suggested to be clinically, pathologically and transcriptionally distinct within CRC [4, 61]. Multi-omics studies aimed at identifying differences between RSCC and LSCC have often targeted a direct comparison between the two tumor sides (e.g. [10]). However, we identify mechanistic differences in proximal and distal tumorigenesis by first comparing it to their respective normal tissues, within a conservatively chosen sample cohort. The results presented here illustrate the power of the study, to discriminate correlations in transcriptional and post-transcriptional regulation, which are likely lost in a direct comparison of the two tumor types, due to compensatory mechanisms in play. For instance, PRAC *(PRAC1)* is a heavily transcribed gene of the prostate, distal colon and rectum in normal physiology [62]. Studies in CRC, comparing RSCC with LSCC, have suggested a suppression of PRAC within proximal tumors [63]. On the contrary, our analysis indicates an overexpression of PRAC (4.6 fc), unique to RSCC, highlighting a possible functional role for PRAC within proximal tumors. Also, the observed upregulation of its adjacent genes (genomic co-ordinates), *HOXB13* and *PRAC2* allude to possible co-regulatory mechanisms in RSCC [64], emphasizing their differential roles in proliferation and tumor growth potential within the two tumor types.

Pathways known to be altered in the pathogenesis of CRC, including WNT and MYC signaling, EMT transition, inflammatory responses (including TNF-α signaling via NFkB), and a suppression of KRAS signaling, were identified in both RSCC and LSCC. Rather than focusing on these traditionally well-known molecular mechanisms, we investigated mechanisms that contribute differentially to the sided-ness of colon cancer. Our findings indicate unique patterns of regulation, specifically within suppressed gene sets of RSCC and LSCC. A detailed analysis suggests a reduced capacity of RSCC to detoxify carcinogens, likely contributing to a genotoxic tumor environment, resulting in a more aggressive phenotype than LSCC. Additionally, increasing evidence of altered metabolism, a critical hallmark of colon cancer, is more evident in RSCC, further lending credence to the link between metabolism and cancer aggressiveness. A larger proportion of differentially regulated miRNAs on the right further support the observation that proximal tumors are more transcriptionally active [65]. Our results are particularly interesting in the context of LSCC, as they establish an enrichment of suppressed chemokine/GPCR signaling markers), at a transcriptional level. When explored within a larger context of dysregulation, using a network theoretic approach, our results highlight a crucial nexus between calcium homeostasis (sensing, mobilization and absorption, e.g. *LPAR1* and *CASR*) and immune/GPCR signaling within LSCC patients, possibly contributing to the differential immune landscape of distal tumors, and their reduced proliferative and metastatic potential.

From a clinical standpoint, several tissue (invasive), and fecal and blood (non-invasive) markers are available for diagnosis and prognosis today [66, 67]. The power of our analysis lies in enabling identification of side and stage specific biomarkers. For instance, Cytokeratin (*KRT7, KRT17* and *KRT20*) are often used as metastasis markers. Notably, we identify additional markers which exhibit preferential expression within distal and proximal tumors and may serve as putative markers for early vs late stage detection. For example, *KRT31* (in early stages) and *KRT40* (in late stages) is expressed in distal tumors alone, while *KRT13* and *KRT15* are expressed in early stages of proximal tumors. We speculate *KRT5*, to be a stage-agnostic marker for RSCC. Likewise, FDA approved serum tumor marker CEA, is encoded by the CEACAM family of genes. Though, *CEACAM1* and *CEACAM7* are similarly upregulated in both LSCC and RSCC, *CEACAM18*- is predominantly upregulated in proximal tumors and may serve as a biomarker for RSCC.

PTR is a key regulatory step between transcription and translation [68]. RBPs and miRNAs are increasingly suggested to exhibit combinatorial regulation of themselves and their target mRNAs [69, 70] making delineating RBP/miRNA-target functional mapping a complex task. We highlight a handful of miRs including miR-29a (in LSCC); miR-155, miR181-d, miR-576 and miR23a (in RSCC), which may serve as side-specific markers of colon tumors, and as suitable candidates for future therapeutic research. Several highly regulated miRs including mir-3607, miR-3677, miR-3622a, miR-885, miR-3620, miR-1295, miR-889, miR-653 with limited prior evidence for roles in colon cancer may serve as interesting targets for future studies, particularly in non-synchronous tumors. We observe that a majority of AS events are mediated by select splicing associated RBPs within both tumors (LSCC and RSCC) including *NOVA1*, *CELF4*, and *RBM20*. Interestingly, *NOVA1* knockdown was recently shown to significantly alter *TERT* transcripts in CRC [71]. Adding another layer to the complexity of PTR are opposing correlational trends between splicing factors and transcripts, suggesting target specific regulation by RBPs. For example, the increased splice-in of *SULT2B1* (AP exon 1) observed in tumors, correlated negatively with the reduced expression of three splicing factors *NOVA1, RBM20* and *CELF4*. Increased splice-in of *CALD1* events within tumors (ES 8.3:9 and AD, 8.3) correlated significantly with the reduced expression of *CELF4, NOVA1*, and *RBM20* in both left and right, suggesting differing impacts of differential regulation by these splicing factors.

Alterations in DNA methylation is an early event in cancer and has been suggested for use in non-invasive diagnosis within CRC [58, 59]. Overall, our analysis of methylation data indicates that hypomethylation is a prominent phenomenon within distal tumors, in contrast to hypermethylation within proximal tumors. Analysis of differentially methylated loci established *FOSL1 and SP1/2/3* binding motifs as highly ranked for hypomethylated and hypermethylated genes respectively, in a side-independent manner within colon cancers. We postulate that *ISX* might serve as a major TF affecting hypermethylation dynamics within LSCC and RSCC. Additionally, our results indicate a positive correlation exists between epigenetic changes and gene expression of several genes, more prominently within LSCC. For instance, *PHACTR3*, a hypermethylated stool biomarker [72] in both tumors, shows a significant positive correlation between hypermethylation and (over) expression only within distal tumors. These instances further highlight a differential role for methylation and underscore alternate mechanisms of expression control within solid tumors, in a side-specific manner.

## Conclusion

In this study, we analyze multi-omics data from colon cancer patients to decipher mechanisms of tumor etiology and progression, which contribute to the side-specificity of colon cancers. We capitalize on publicly available data to identify distinctions in molecular signatures at an epigenetic (methylation), transcriptional (stage specific and stage-agnostic) and post-transcriptional levels (miRNA, alternative splicing mediated by RNA binding proteins) between right and left colon cancers. We show signatures associated with tumor aggressiveness arise from the genotoxic environment in RSCC and those with suppressed chemokine/immune response are more prevalent in LSCC. Pronounced dysregulation of miRNAs and RBPs- two well-known post-transcriptional regulators, on the right further strengthen the argument of a transcriptionally hyperactive and diverse RSCC. In addition to the differential methylation patterns, a surprising number of positive correlations (between differential methylation and expression) in both RSCC and LSCC underscore alternate mechanisms for expression control within colon tumors. Differences in functional mechanisms described here emphasize the molecular heterogeneity of colon cancers. Future experimental validation of the molecular players identified here, specifically within non-synchronous tumors, will influence the efficacies of existing and future diagnostic (e.g. biomarker identification), prognostic (patient stratification and recurrence) and therapeutic (e.g. molecular) interventions.

## Supplementary information

**Additional file 1.** An excel file containing all supplementary tables highlighted with the manuscript as individual sheets.

**Additional file 2: Figure 1.** RSCC specific miR-mRNA interaction network clustered via MCL clustering. Validated interactions between differentially expressed genes and miRs were extracted from the miR-TAR database. The network was clustered on mRNA-miRNA co-expression strength using MCL clustering. Node color: Blue- downregulated, red- upregulated, greencommonly regulated miR and orange- uniquely regulated miR. miR label color: redupregulated, blue- downregulated. **Figure 2** LSCC specific miR-mRNA interaction network clustered via MCL clustering. Validated interactions between differentially expressed genes and miRs were extracted from the miR-TAR database. The network was clustered on mRNA-miRNA co-expression strength using MCL clustering. Node color: Blue- downregulated, red- upregulated, green- commonly regulated miR and orange- uniquely regulated miR. miR label color: red- upregulated, bluedownregulated. **Figure 3** - Correlation network of splicing factors and AS events- the correlation network for left and right tumors captures the correlation between splicing factors (differentially regulated RBPs) and sigAS events occurring in each side. Blue edges indicated anti-correaltion, red- correlation. Pink nodes are the splicing factors, grey nodes indicate sigAS events. The shapes correspond to each of the six events identified and presented in legend within figure.

## Data Availability

The datasets analyzed in the current study are all publicly available in the Genomic data commons (GDC) repository, [https://portal.gdc.cancer.gov/projects/TCGA-COAD]. No explicit permission was required to download level 3 data for TCGA-COAD from Firebrowse.

## Abbreviations

5-HT: 5-hydroxytryptamine
AA: Arachidonic acid
AAs: Alternate acceptor sites
AD: Alternate donor sites
AP: Alternate promoter
AS: Alternative splicing
AT: Alternate terminator
CA: Carbonic anhydrases
CRC: Colorectal cancer
CIMP: CpG island methylation phenotype
DEGs: Differentially expressed genes
DEMs: Differentially expressed miRNAs
DMPs: Differentially methylated probes
EETs: Epoxyeicosatrienoic acids
EMT: Epithelial-to-mesenchymal transition
ES: Exon skip
GI: Gastrointestinal
LSCC: Left-sided colon cancers
miR: micro-RNAs
MSI: Microsatellite instability
ME: Mutually exclusive exons
NCCN: National Comprehensive Cancer Network
OS: Overall survival
OXOPHOS: mitochondrial oxidative phosphorylation
PPI: Protein-protein interactions
PTR: Post-transcriptional regulation
RBP: RNA binding protein
RI: Retained intron
RSCC: Right-sided colon cancers
SEER: Surveillance, Epidemiology and End Result
SERT: Serotonin-selective reuptake transporter
TCGA: The Cancer Genome Atlas
TF: Transcription factor
UGT: UDP-glucuronosyltransferase
