# Supplementary material for: Right and left-sided colon cancers - specificity of molecular mechanisms in tumorigenesis and progression
Source: BMC Cancer. 2020 Apr 15;20:317. doi: 10.1186/s12885-020-06784-7 (PMC7161305; doi:10.1186/s12885-020-06784-7)
Supplement: Supplementary file 2 — Additional file 2: Figure 1. RSCC specific miR-mRNA interaction network clustered via MCL clustering. Validated interactions between differentially expressed genes and miRs were extracted from the miR-TAR database. The network was clustered on mRNA-miRNA co-expression strength using MCL clustering. Node color: Blue- downregulated, red- upregulated, greencommonly regulated miR and orange- uniquely regulated miR. miR label color: redupregulated, blue- downregulated. Figure 2 LSCC specific miR-mRNA interaction network clustered via MCL clustering. Validated interactions between differentially expressed genes and miRs were extracted from the miR-TAR database. The network was clustered on mRNA-miRNA co-expression strength using MCL clustering. Node color: Blue- downregulated, red- upregulated, green- commonly regulated miR and orange- uniquely regulated miR. miR label color: red- upregulated, bluedownregulated. Figure 3 - Correlation network of splicing factors and AS events- the correlation network for left and right tumors captures the correlation between splicing factors (differentially regulated RBPs) and sigAS events occurring in each side. Blue edges indicated anti-correaltion, red- correlation. Pink nodes are the splicing factors, grey nodes indicate sigAS events. The shapes correspond to each of the six events identified and presented in legend within figure. [file 12885_2020_6784_MOESM2_ESM.pdf]

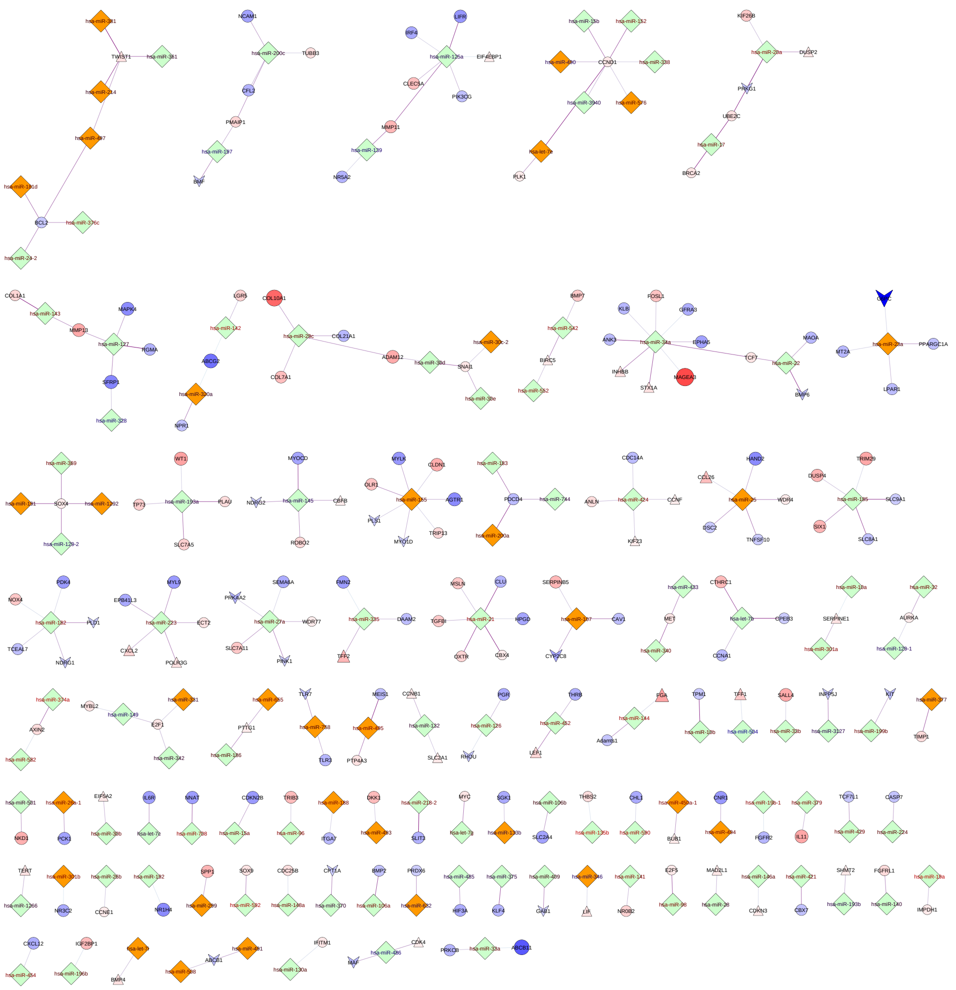

Figure 1 RSCC specific miR-mRNA interaction network clustered via MCL clustering. Validated interactions between differentially expressed genes and miRs were extracted from the miR-TAR database. The network was clustered on mRNA-miRNA co-expression strength using MCL clustering. Node color: Blue- downregulated, red- upregulated, green- commonly regulated miR and orange- uniquely regulated miR. miR label color: red- upregulated, blue- downregulated.

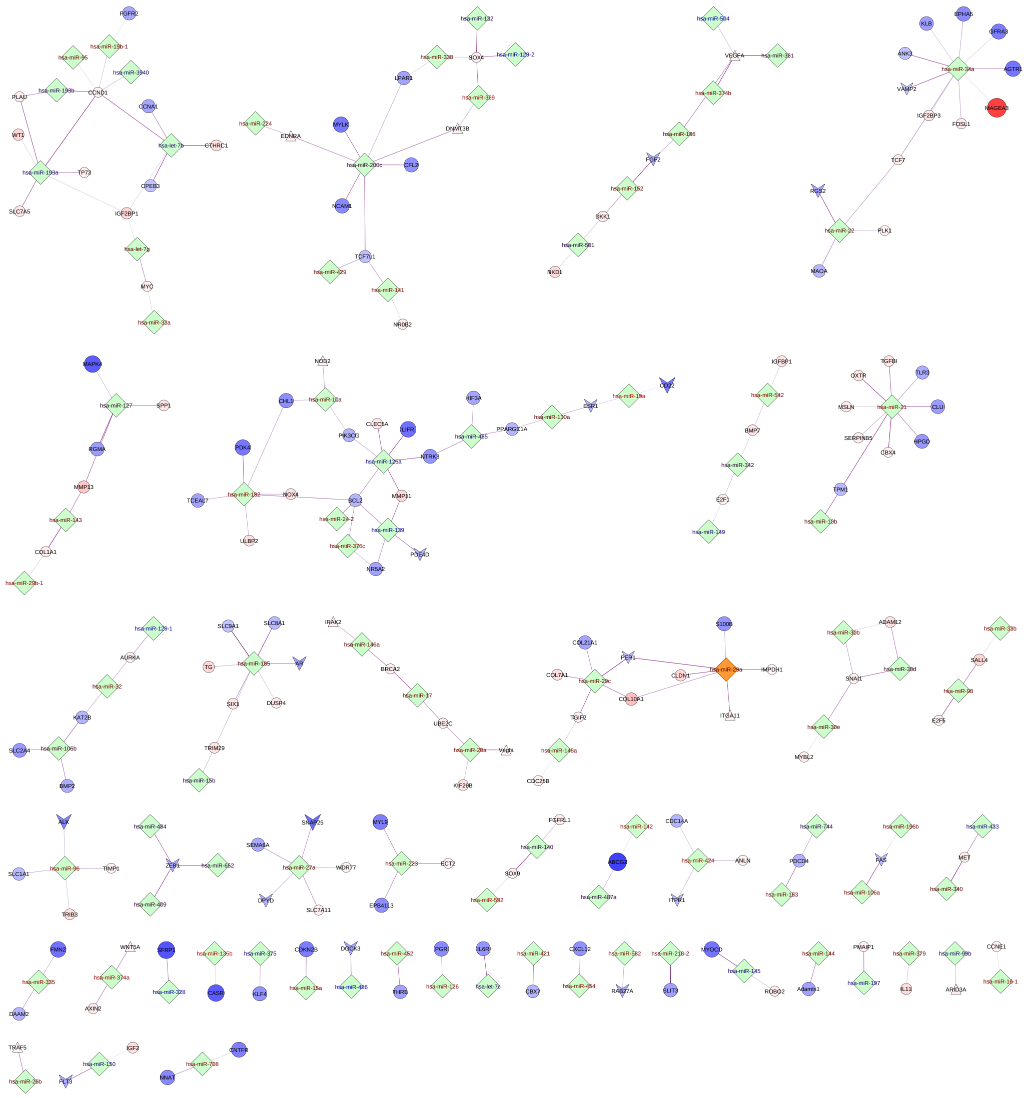

Figure 2 LSCC specific miR-mRNA interaction network clustered via MCL clustering. Validated interactions between differentially expressed genes and miRs were extracted from the miR-TAR database. The network was clustered on mRNA-miRNA co-expression strength using MCL clustering. Node color: Blue- downregulated, red- upregulated, green- commonly regulated miR and orange- uniquely regulated miR. miR label color: red- upregulated, blue- downregulated.

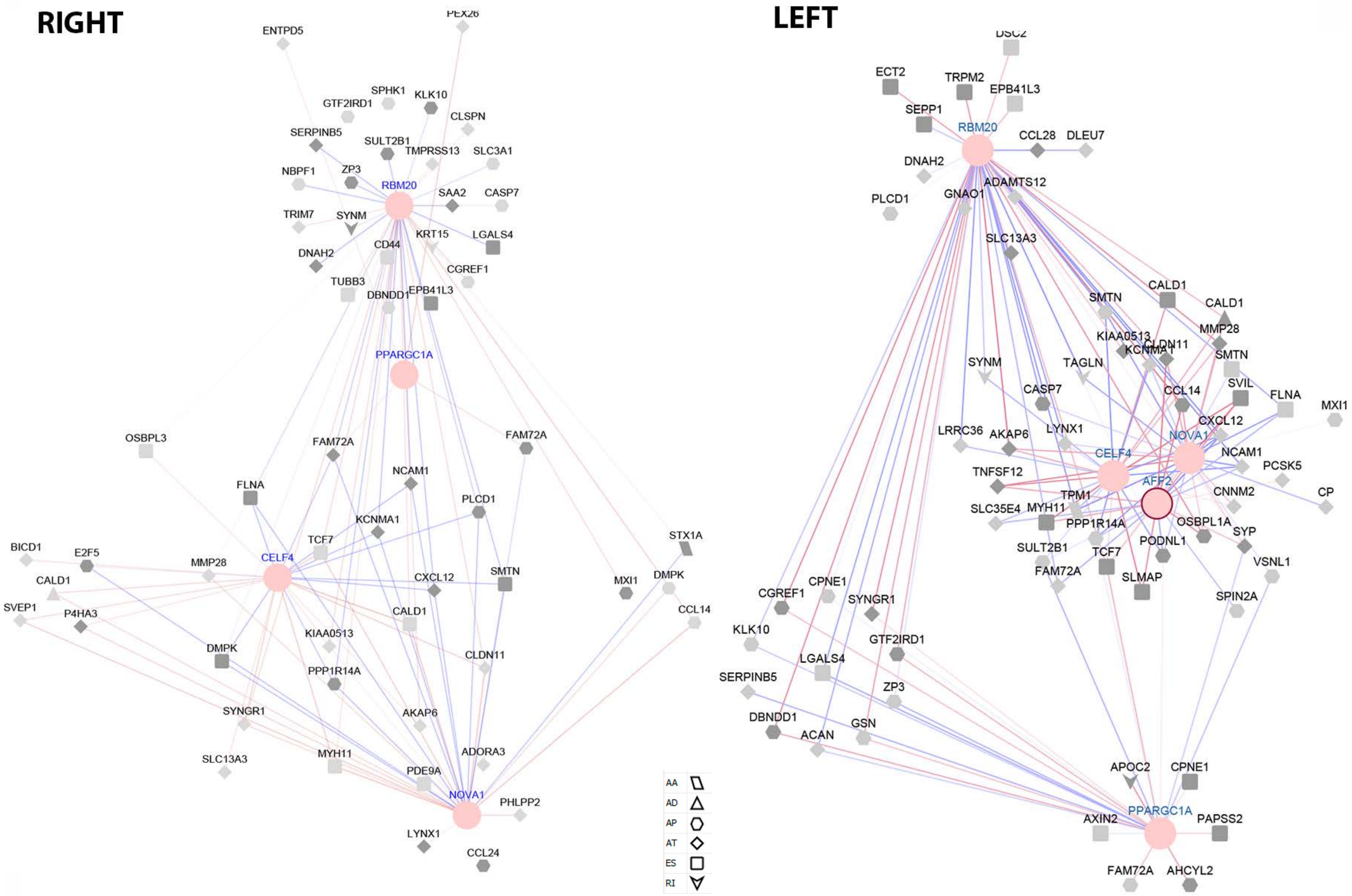

Figure 3 - Correlation network of splicing factors and AS events- the correlation network for left and right tumors captures the correlation between splicing factors (differentially regulated RBPs) and sigAS events occurring in each side. Blue edges indicated anti-correealtion, red- correlation. Pink nodes are the splicing factors, grey nodes indicate sigAS events. The shapes correspond to each of the six events identified and presented in legend within figure.
